# Refolding of *β*-Stranded Class I Chitinases of *Hippophae rhamnoides* Enhances the Antifreeze Activity during Cold Acclimation

**DOI:** 10.1371/journal.pone.0091723

**Published:** 2014-03-13

**Authors:** Ravi Gupta, Renu Deswal

**Affiliations:** Molecular Plant Physiology and Proteomics Laboratory, Department of Botany, University of Delhi, Delhi, India; Aligarh Muslim University, India

## Abstract

Class I chitinases hydrolyse the *β*-1,4-linkage of chitin and also acquire antifreeze activity in some of the overwintering plants during cold stress. Two chitinases, HrCHT1a of 31 kDa and HrCHT1b of 34 kDa, were purified from cold acclimated and non-acclimated seabuckthorn seedlings using chitin affinity chromatography. 2-D gels of HrCHT1a and HrCHT1b showed single spots with pIs 7.0 and 4.6 respectively. N-terminal sequence of HrCHT1b matched with the class I chitinase of rice and antifreeze proteins while HrCHT1a could not be sequenced as it was N-terminally blocked. Unlike previous reports, where antifreeze activity of chitinase was cold inducible, our results showed that antifreeze activity is constitutive property of class I chitinase as both HrCHT1a and HrCHT1b isolated even from non-acclimated seedlings, exhibited antifreeze activity. Interestingly, HrCHT1a and HrCHT1b purified from cold acclimated seedlings, exhibited 4 and 2 times higher antifreeze activities than those purified from non-acclimated seedlings, suggesting that antifreeze activity increased during cold acclimation. HrCHT1b exhibited 23–33% higher hydrolytic activity and 2–4 times lower antifreeze activity than HrCHT1a did. HrCHT1b was found to be a glycoprotein; however, its antifreeze activity was independent of glycosylation as even deglycosylated HrCHT1b exhibited antifreeze activity. Circular dichroism (CD) analysis showed that both these chitinases were rich in unusual *β*-stranded conformation (36–43%) and the content of *β*-strand increased (∼11%) during cold acclimation. Surprisingly, calcium decreased both the activities of HrCHT1b while in case of HrCHT1a, a decrease in the hydrolytic activity and enhancement in its antifreeze activity was observed. CD results showed that addition of calcium also increased the *β*-stranded conformation of HrCHT1a and HrCHT1b. This is the first report, which shows that antifreeze activity is constitutive property of class I chitinase and cold acclimation and calcium regulate these activities of chitinases by changing the secondary structure.

## Introduction

Plant chitinases are classified into five classes (I to V) on the basis of their primary structure. These five classes of chitinase belong to either glycoside hydrolase (GH) 18 or GH 19 family. Class I chitinase belongs to GH 19 family and contain an N-terminal chitin binding domain and a C-terminal catalytic domain. Besides, hydrolysing the *β*-1,4-glycosidic bond of chitin, chitinases of some overwintering plants also exhibit antifreeze activity during cold stress. Antifreeze proteins (AFPs) are a class of polypeptides which adsorb to ice crystals and retard their growth by preventing the addition of water molecules to growing ice crystal planes. AFPs prevent ice recrystallization, a process which leads to the formation of larger ice crystals from the smaller ones. This reduces the physical injury caused by the larger ice crystals present in the apoplast. AFPs increase survival of plants by modifying ice crystal growth, decreasing the freezing temperature, inhibiting ice recrystallization, slowing down the ice migration in tissue and cryo-protecting the metabolic enzymes [1], [2]. Out of six AFPs purified in winter rye, two of 35 kDa and 28 kDa were identified as class I and class II chitinases respectively by N-terminal sequencing and western blot analysis [3]. Expression analysis of *CHT9* (class I chitinase) and *CHT49* (class II chitinase) transcripts in winter rye showed their induction during cold acclimation and recombinant proteins overexpressed in *Escherichia coli* showed antifreeze activity [4]. In bromograss suspension cells, a cold responsive chitinase gene *BiCHT1* was identified which was homologous to winter rye *CHT9*. *BiCHT1* expression was induced by low temperatures but, when it was overexpressed in either *E. coli* or bromograss cells, no antifreeze activity was observed until the culture medium was incubated at 4°C, suggesting that BiCHT1 exhibits antifreeze activity only after cold treatment [5]. Recently, a class I chitinase isolated from *Chimonanthus praecox* L. (wintersweet) petals was shown to exhibit high antifreeze activity, suggesting that class I chitinases from dicots also exhibit antifreeze activity [6]. In contrast, class I chitinase of *Vitis vinifera*, lacks antifreeze activity, although it was able to cryoprotect the lactate dehydrogenase during freezing and thawing [7]. Besides cold stress, class I chitinase also accumulates in winter rye apoplast during drought stress and exhibits antifreeze activity. However, class I chitinase which accumulates in response to salicylic acid and snow mould infection lacks antifreeze activity [8]. These results showed that same protein exhibits antifreeze activity in some environmental conditions and not in others. What actually regulates the acquisition of antifreeze activity in class I chitinase is still unclear.

Recently, antifungal class I and class III chitinases were isolated from seabuckthorn [9]. Induction of seabuckthorn chitinase both at transcript and protein levels in cold stress has already been shown. Freezing stress induced 8 fold accumulation of chitinase transcripts and 22 fold accumulation of chitinase protein was shown in seabuckthorn [10],[11]. Almost three fold higher accumulation of protein in comparison with transcripts suggests the role of post transcriptional and/or post translational modifications (PTMs) during cold stress. Not much is known about the PTMs of class I chitinase. Few studies which have been performed showed glycosylation and hydroxylation of class I chitinase in different plants. Class I chitinase of pineapple is glycosylated [12], however, no class I chitinase, exhibiting antifreeze activity is found to be glycosylated, therefore, regulation of antifreeze activity of class I chitinase by glycosylation is not investigated. Although, both class I chitinase of tobacco and winter rye are hydroxylated [13],[14], tobacco chitinase does not exhibit antifreeze activity while winter rye chitinase has antifreeze activity, suggesting no correlation of hydroxylation with antifreeze activity [4]. It was shown that winter rye chitinase refolds during freezing and thawing in presence of 20 mM CaCl_2_ leading to enhancement of its hydrolytic activity [15] while class I chitinase of bromograss did not respond to calcium even after 1–7 days of incubation [5].

The objective of this study was to ascertain whether seabuckthorn chitinase also has antifreeze activity or not and if it exhibits antifreeze activity, how is it regulated? For this, class I chitinase was purified from both non-acclimated and cold acclimated seedlings and tested for the hydrolytic and antifreeze activities. Circular Dichroism was used to predict any change in the secondary structure. The results obtained from the current study established for the first time that antifreeze activity is constitutive property of class I chitinases which increases during cold acclimation. Besides, we have also shown that class I chitinases undergo conformational changes during cold acclimation and in presence of calcium which might be responsible for the increased antifreeze activity of these chitinases.

## Materials and Methods

### Plant Growth Conditions and Cold Stress Treatment

*Hippophae rhamnoides* (seabuckthorn) seeds were isolated from berries collected from the wild, along the riverside of Bhaga River from Keylong, Lahaul and Spiti valley of Himachal Pradesh. As seabuckthorn is not a cultivated crop and is neither endangered nor protected plant as per Botanical Survey of India (http://164.100.52.111), no specific permissions were required for the sample collection. Seeds were washed, dried and stored in a desiccator at RT and were germinated as described previously [11]. For cold stress treatment, 20 days old seedlings were transferred at 4°C with 35 µmol/m^2^/s, 8 h light/16 h dark for 3 weeks. For control, seedlings were maintained at 25°C with 270 µmol/m^2^/s, 16 h light/8 h dark.

### Extraction of the Apoplastic Proteins

Apoplastic proteins were isolated following Gupta and Deswal [11] by a modified vacuum infiltration protocol. In brief, seedlings were cut into small segments and incubated under vacuum in a buffer containing 20 mM ascorbic acid and 20 mM calcium chloride for 30 min. Apoplastic proteins were isolated by centrifuging the segments at 4000 g for 10 min. Isolated proteins were subjected to acetone precipitation and quantified using Bradford’s method [16].

### Chitin Affinity Chromatography

High affinity of class I chitinase with chitin was used for its single step purification by chitin affinity chromatography. Colloidal chitin was prepared from chitin flakes following Kang et al. [17] and chitinase was purified using a modified procedure [3],[18]. In brief, colloidal chitin was added to the protein extract until the O.D at 590 reached 0.8–0.9 and the mixture was incubated overnight at 4°C with constant stirring. Mixture was then centrifuged at 16000 g for 5 min at 4°C and the supernatant was removed (Flow through). Colloidal chitin was then washed four times with ice cold 20 mM ammonium bicarbonate (ABC) pH 8.0 and twice with 10 mM acetic acid. Chitinase was then eluted from the chitin column either using 20 mM or 250 mM acetic acid pH 3.0 for 30 min at ice and neutralized immediately. The purity of purified protein was checked on SDS-PAGE and 2-DGE [19].

### Two Dimensional Gel Electrophoresis

For 2-DE, 70 µg of purified chitinase was loaded on a nonlinear IPG strip, pH 3–10 by rehydration loading overnight at 20°C. IEF was performed on IttanIPGphore (GE Healthcare) as described previously [11]. After IEF, the strips were equilibrated using equilibration buffer (6 M urea, 50 mM Tris pH 8.8, 30% glycerol, 2% SDS, and 0.002% BPB) containing 1% DTT as the first step and then alkylated by 2.5% iodoacetamide as the second step. For the second dimension, proteins were resolved on 15% SDS-PAGE using Hoefer SE 600 Ruby (GE Healthcare). The gels were silver stained [20] and pI and molecular weight of the spots were calculated using ImageMaster2DPlatinum software ver. 6.0 (GE Healthcare).

### N-terminal Sequencing

For N-terminal sequencing, purified chitinases were separated on SDS-PAGE and electroblotted onto a PVDF membrane. After transfer in CAPS buffer pH 8, the two separate bands of chitinase were excised from the membrane, washed with methanol and subjected to amino-terminal sequencing using automated gas phase sequencer (Applied Biosystems 491-A Procise 4.0).

### Detection of Glycoprotein by Affinoblotting

Detection of glycoproteins was done using affino-blotting method using Concanavalin-A and horseradish peroxidase. After the resolution on SDS-PAGE, the proteins were transferred to nitrocellulose membrane and then stained with Ponceau-S (Sigma-Aldrich) to visualize the protein transfer. After transfer and protein visualization, the membrane was preincubated in 3% BSA in phosphate buffered saline (PBS) for 1 h at 37°C to prevent nonspecific binding. The membrane was then immersed overnight in 50 mL Con-A solution (10 µg/mL, Sigma-Aldrich) containing 50 mM MnCl_2_, 100 mM CaCl_2,_ 100 mM MgCl_2_ and 0.5% Triton-X 100 in PBST (PBS containing 0.05% Tween-20). Washing was done in PBST for 30 min and then the membrane was incubated with a solution of horseradish peroxidase (50 µg/mL, Sigma-Aldrich) for 1 hr. The blot was then incubated in dark with 3.4 mM 4-chloro-1-napthol (Sigma-Aldrich) in 100 mM Tris pH 7.5 containing 0.3% H_2_O_2_. After appearance of bands, the reaction was stopped with water. For deglycosylation, purified HrCHT1b was subjected to deglycosylation using both N-glycosidase (PNGase F, Sigma-Aldrich), O-glycosidase (New England Biolabs) and their combination following manufacturer’s instructions. In addition, a chemical method using trifluromethanesulphonic acid was also used for the deglycosylation of HrCHT1b [21].

### Chitinase Hydrolytic Activity Assays

Chitinase activity was determined using colloidal chitin as substrate by measuring the production of N-acetylglucosamine (GlcNAc) using a previously published method [22]. The reaction mixture of 500 µL was prepared which consisted of 100 µL enzyme and 200 µL of 2% colloidal chitin in 50 mM sodium acetate buffer pH 5.0. The reaction mixture was incubated at 37°C for 30 min, and then the reaction was terminated by heating in boiling water for 15 min. The reducing end group produced was measured colorimetrically by potassium ferricyanide assay [23]. The reaction mixture was incubated with 1.0 mL potassium ferricyanide reagent in boiling water for 15 min, and then subjected to spectrophotometry measurement at 410 nm. One unit of chitinase activity was defined as capable of releasing reducing ends corresponding to 1 µg of GlcNAc per minute. Assays were performed in three biological replicate with each assay performed in triplicate. Results of the chitinase activity assays were subjected to Student’s t-test. A value of *p*<0.05 was considered statistically significant.

### Antifreeze Activity Analysis

For antifreeze activity, ice crystal morphologies were observed using phase contrast microscope (Nikon eclipse 80*i*) along with a nanoliter osmometer (Otago osmometers). Bovine Serum Albumin (BSA), a non-antifreeze protein, was used as a negative control and 20 mM ABC (buffer) was used as blank. Ice recrystallization inhibition (IRI) assays were performed using sucrose sandwich splat assay as described earlier [11]. For the quantification of the IRI activity, the diameters of the ice crystals in each image were measured using Image J and endpoint of IRI was calculated. For analysing the effect of calcium, purified chitinases were incubated with different concentration of CaCl_2_ (0–0.4 mM) and analysed for antifreeze and hydrolytic activities. All experiments were performed in three technical and three biological replicates. Statistical analysis was performed by one-way ANOVA and post hoc Tukey HSD test, applied for multiple paired comparisons. A value of *p*<0.05 was considered statistically significant.

### Circular Dichroism

For analysing the secondary structures, purified chitinases were filtered through 0.2 µM filter to remove protein aggregates and far-UV (190–250 nm) spectra were acquired using a spectropolarimeter J-815 (Jasco International, Tokyo, Japan) connected to a Peltier temperature controlled sample holder. For each experiment, three spectra were acquired and averaged at a concentration of 100 µg/mL with 1-mm path length and a 1-nm bandwidth, at a scanning rate of 50 nm/min at 20°C. For analysing the unfolding kinetics of purified chitinase, purified protein was heated at every 2°C interval from 10–94°C using the Peltier device and spectra were acquired in triplicates and averaged. K2D3 server was used for the quantitative analysis of the CD results [24].

## Results and Discussion

### Purification of Chitinase from Cold Acclimated and Non-acclimated Seedlings

**Figure 1:**
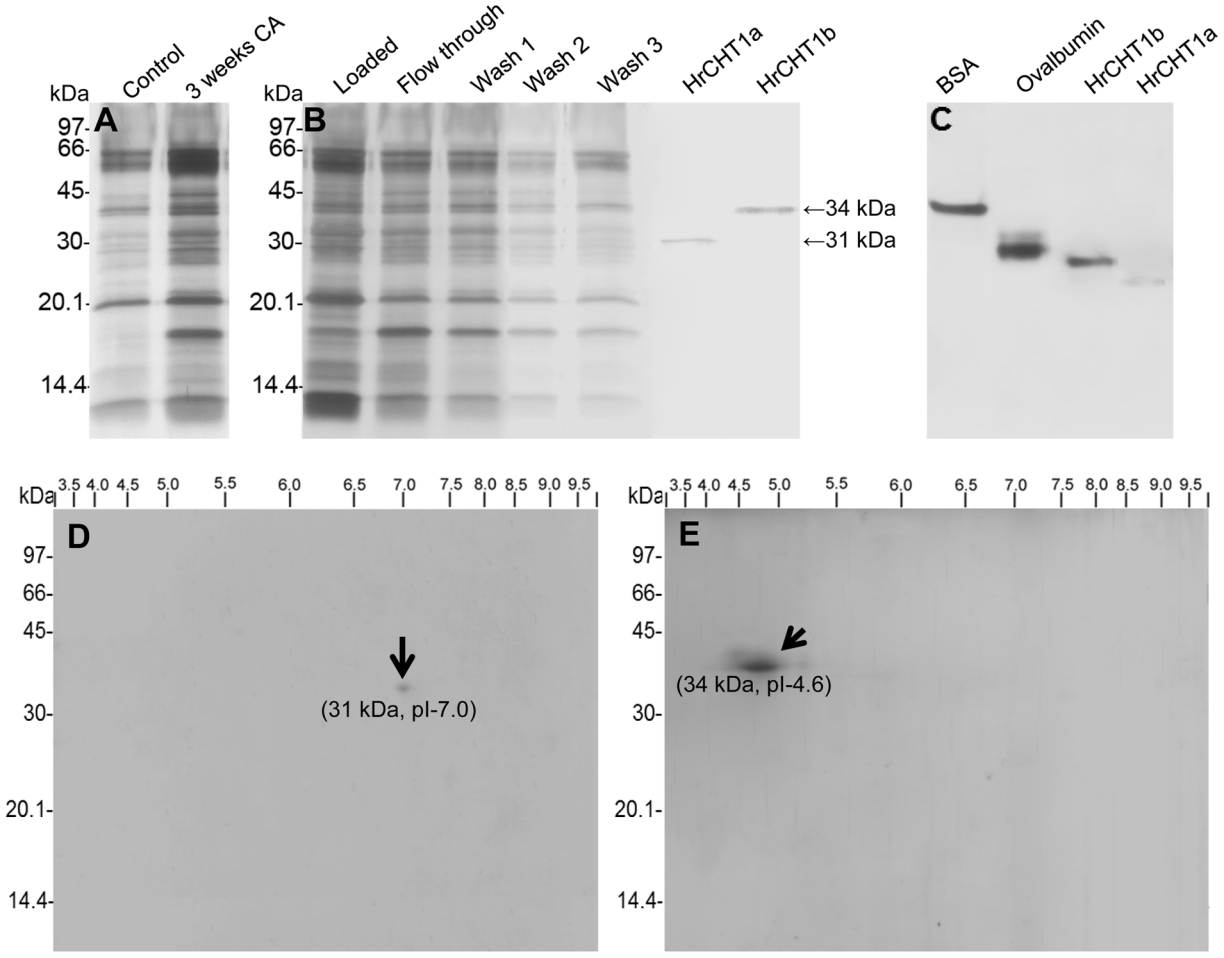
Purification of HrCHT1a and HrCHT1b. (A) Silver stained 15% SDS-PAGE gel showing apoplastic proteins isolated from non-acclimated and 3 weeks cold acclimated seedlings. (B) SDS-PAGE and (C) 10% native gel showing purified class I chitinase from 3 weeks cold acclimated apoplastic extracts using chitin affinity chromatography. (D) Two dimensional gel electrophoresis of HrCHT1a and HrCHT1b (E). Purified HrCHT1a and HrCHT1b (70 µg) were loaded on 3–10 non-linear IPG strips and subjected to iso-electric focusing. After IEF, proteins were resolved on 15% SDS-PAGE for the second dimension.

In our previous study, we observed 20 fold accumulation of chitinase at –5°C in shoot apoplast which indicated its probable role in cold stress tolerance. In addition, an antifreeze protein, homologous to polygalacturonase inhibitor protein, was purified by ice adsorption chromatography. Analysis of the unbound fraction of the ice adsorption chromatography also exhibited antifreeze activity which suggested the presence of other antifreeze proteins in seabuckthorn apoplast [11]. In winter rye, two chitinases with antifreeze activities were identified of which one was class I chitinase [3]. Therefore, this study was conducted to know if class I chitinases with antifreeze activity exist in seabuckthorn or not. Apoplastic proteins from non-acclimated and 3 weeks cold acclimated seedlings were used to purify the class I chitinase using chitin affinity chromatography. Initially, 20 mM acetic acid was used to elute the chitin binding protein from the chitin affinity column, which led to the purification of a 31 kDa polypeptide (HrCHT1a). To check the amount of chitin binding protein which was not eluted from the column, colloidal chitin slurry after elution with 20 mM acetic acid, was reconstituted in 500 µL SDS-PAGE sample buffer, boiled and loaded on the gel. Surprisingly, a very thick band of 34 kDa was observed in this slurry fraction. Repeated elution with 20 mM acetic acid failed to elute this 34 kDa chitin binding protein from the chitin affinity column. Therefore, higher concentrations of acetic acid (25–250 mM) were tried to elute this 34 kDa protein. Step elution of chitin binding proteins using 25, 50 and 100 mM acetic acid, led to the elution of both 31 kDa and 34 kDa polypeptide (Figure S1). To elute 34 kDa polypeptide (HrCHT1b) separately, still higher concentration *i.e* 250 mM of acetic acid was used (Figure 1B). Native-PAGE of these chitin binding polypeptides showed these to be the monomers (Figure 1C). Densitometric scanning of SDS-PAGE gel showed that the amount of purified HrCHT1a to be ∼7.2 times lesser than HrCHT1b. There could be two possibilities: either the amount of HrCHT1a was endogenously low or it could have lower chitin binding affinity and the current protocol used for its purification was not able to purify it completely. On the basis of the results obtained in this study, the second possibility seems correct. HrCHT1a was eluted at very low concentration of acetic acid, showing its weak binding with chitin affinity column while HrCHT1b was eluted at higher concentration of acetic acid suggesting its strong binding to chitin. In addition, analysis of chitinase activity showed that HrCHT1a and HrCHT1b exhibited 563.03 units/mg and 694.35 units/mg activities respectively, further suggesting that HrCHT1b was more potent than HrCHT1a. Chitin affinity chromatography from non-acclimated seedlings also resulted in purification of the same polypeptides. However, these chitinases showed 31.79% and 42.65% higher hydrolytic activities than cold acclimated chitinases (742.82 and 990.29 units/mg for HrCHT1a and HrCHT1b respectively), suggesting a decline in the hydrolytic activity during cold acclimation.

**Figure 2:**
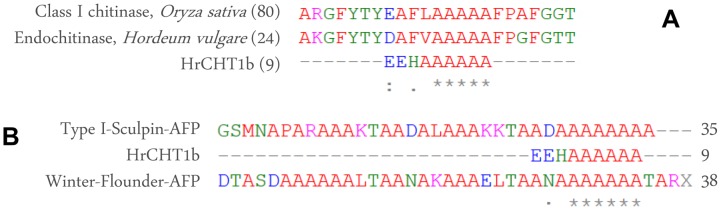
N-terminal sequence of HrCHT1b. Alignment of N-terminal HrCHT1b sequence with other chitinases (A) and antifreeze proteins (B).

Using two dimensional gel electrophoresis, the pIs of the HrCHT1a and HrCHT1b was determined to be 7.0 and 4.6 respectively (Figure 1D–E). In pineapple also, chitin affinity chromatography resulted in the purification of two class I chitinases of 33 and 39 kDa, with almost similar pIs of 7.9 and 4.6 [12]. MALDI-TOF-MS failed to identify HrCHT1a and HrCHT1b, suggesting either these to be the novel proteins or are missing as not many proteins of seabuckthorn are submitted in the database. Therefore, these were subjected to N-terminal sequencing by Edman degradation. HrCHT1a could not be identified as it was N-terminally blocked while first 9 amino acids of HrCHT1b were identified as “**EEHAAAAAA**”. This sequence on blast search in pdb database (Protein Data Bank), matched with class I chitinase of *Oryza sativa* and two other chitinases as shown in Figure 2A. In addition, the N-terminal sequence of HrCHT1b matched with winter flounder antifreeze protein and recombinant type I Sculpin antifreeze protein. In NCBInr database, the sequence of HrCHT1b matched with putative antifreeze glycoprotein [*Streptomyces viridochromogenes* Tue 57] (Figure 2B). These results suggested that HrCHT1b was a class I chitinase with sequence similarities with antifreeze glycoprotein (AFGP) and type I AFP of fishes. However, neither the N-terminal sequence of HrCHT1b nor the sizes of HrCHT1b and HrCHT1a matched with two previously submitted seabuckthorn class I chitinase sequences of accession no. AFC88126.1 and ADP68560.1 in NCBI, suggesting these to be the novel isoforms. In rice, five isoforms of class I chitinases are present in database (NCBInr) suggesting possible existence of multiple isoforms of class I chitinase in other plants also. Size of class I chitinase exhibiting antifreeze activity varies from 32 to 35 kDa in different plants. It is of 35 kDa in winter rye, 32 kDa in bromograss and 33 kDa in *C. praecox*.

**Figure 3:**
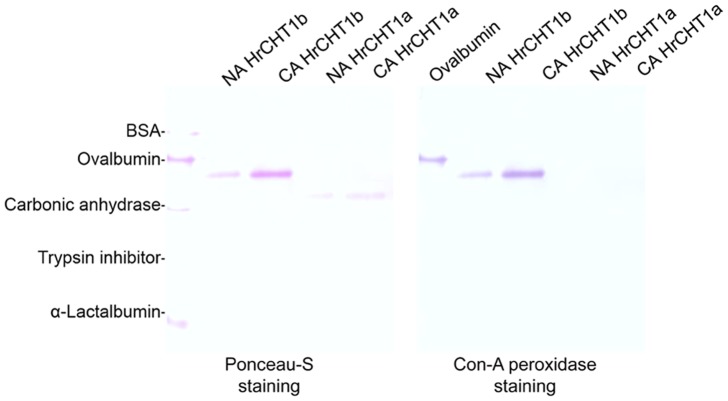
Glycosylation analysis of HrCHT1a and HrCHT1b. Figure showing Ponceau-S staining (A) and Con-A peroxidase staining (B) of HrCHT1a and HrCHT1b purified from non-acclimated and cold acclimated seedlings to detect their glycosylation.

As sequence of HrCHT1b matched with the antifreeze glycoprotein, glycosylation of purified chitinases was checked using Con-A-peroxidase staining. HrCHT1a and HrCHT1b, isolated from both non-acclimated and cold acclimated seedlings, were checked for their glycosylation. Interestingly, HrCHT1b, purified from both non-acclimated and cold acclimated seedlings showed purple colour while HrCHT1a did not stain, showing HrCHT1b to be a glycoprotein. Ovalbumin (positive control) stained purple but BSA (negative control) did not, showing specificity of the reaction (Figure 3). HrCHT1b purified from both non-acclimated and cold acclimated seedlings showed similar extent of glycosylation, showing that glycosylation of HrCHT1b was not affected by cold stress.

### Antifreeze Activity of HrCHT1a and HrCHT1b Increases during Cold Acclimation

**Figure 4:**
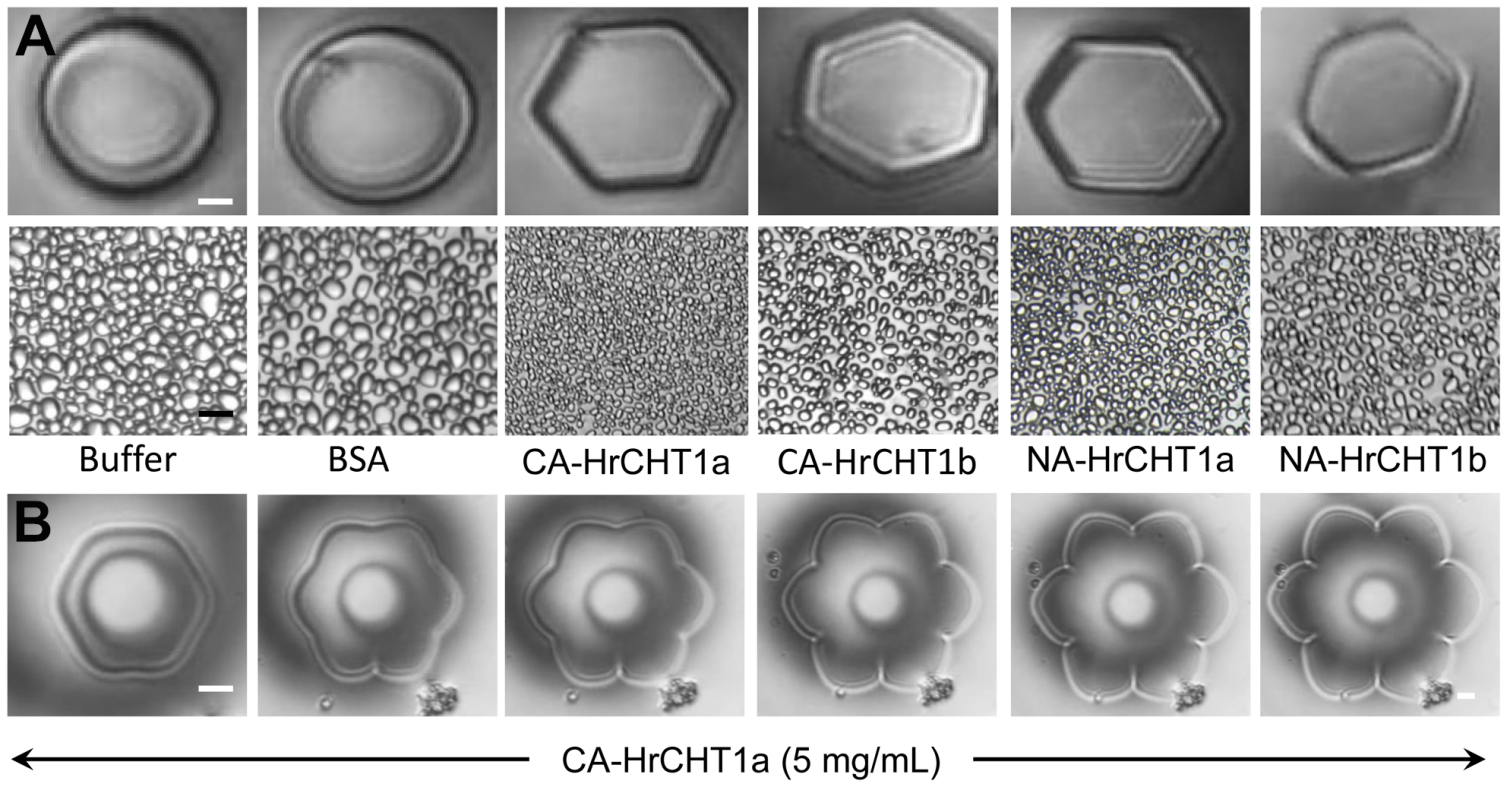
Analysis of antifreeze activity in HrCHT1a and HrCHT1b purified from cold acclimated and non-acclimated seedlings. (A) Upper panel-ice crystal morphology, analysed by phase contrast microscope attached with nanoliter osmometer. Lower Panel-Ice recrystallization inhibition assays as observed by sucrose sandwich splat assay. BSA was used a negative control which did not exhibit antifreeze activity. Magnification bar in ice crystal morphology shows 10 µm, while in splat assay it represents 50 µm. Error bars show standard deviation from five replicates. (B) Ice crystal morphologies showing different stages of formation of flower shaped ice crystal with 5 mg/mL HrCHT1a.

To analyse the antifreeze activity of HrCHT1a and HrCHT1b, ice crystal morphology was analysed using phase contrast microscopy attached with a nanoliter osmometer and ice recrystallization inhibition assays were performed with sucrose sandwich splat assays. In the absence of AFPs, ice crystals grow as circular discs while in the presence of AFPs, flat hexagonal or flower shaped ice crystals are formed. BSA, a non-antifreeze protein, showed formation of circular, disc shaped ice crystal and did not inhibit ice recrystallization while both HrCHT1a and HrCHT1b, purified from cold acclimated seedlings, showed formation of hexagonal ice crystals, indicating association of antifreeze activity (Figure 4A). In addition, HrCHT1a also showed flower shaped ice crystals at ∼5 mg/mL (Figure 4B). Quantification of antifreeze activity was done by calculating the end point of IRI, which is defined as the lowest concentration of antifreeze protein which can inhibit the ice recrystallization. Lower values of endpoint of IRI represent higher antifreeze activity. Endpoint of IRI for HrCHT1a and HrCHT1b, isolated from cold acclimated seedlings, were calculated as 15 µg/mL and 60 µg/mL respectively, showing that the HrCHT1a exhibits 4 times higher antifreeze activity than HrCHT1b. After confirming the antifreeze activity of HrCHT1a and HrCHT1b, next aim was to analyse the factors/conditions which are responsible for the acquisition of the antifreeze activity during cold stress. Therefore, HrCHT1a and HrCHT1b, purified from non-acclimated seedlings, were also tested for the antifreeze activity. Surprisingly, both the chitinases, even from non-acclimated seedlings, exhibited antifreeze activity and modified ice crystal growth, indicating class I chitinase in non-acclimated seedlings also had antifreeze activity. However, the antifreeze activities were lower in these chitinases in comparison with those purified from cold acclimated seedlings. Endpoint of IRI for HrCHT1a and HrCHT1b, isolated from non-acclimated seedlings, were calculated as 60 µg/mL and 120 µg/mL respectively, indicating an enhancement in the antifreeze activity of these chitinases during cold acclimation. These results clearly showed that the antifreeze activity is constitutive property of seabuckthorn class I chitinases unlike previous reports which suggested that class I chitinase acquire antifreeze activity during stress conditions. In winter rye, class I chitinase overexpressed in *E. coli*, exhibited antifreeze activity when induced by IPTG at 25°C without any cold acclimation [4], indicating that class I chitinase of winter rye may also have constitutive antifreeze activity. However, same chitinase produced by salicylic acid treatment [25] or snow-mould infection [26] lacked antifreeze activity in winter rye. Moreover, class I chitinase of bromograss (BiCHT1) did not exhibit antifreeze activity when purified from non-acclimated culture cells [5]. These results indicate that some post translational event either any PTM or refolding of chitinase during stress conditions regulate its antifreeze activity.

HrCHT1b was found to be glycosylated. As there was not much difference in the sizes of HrCHT1a and HrHT1b, it was possible that the HrCHT1a could be the deglycosylated form of HrCHT1b. Therefore, an attempt was made to deglycosylate HrCHT1b using both N-glycosidase and O-glycosidase. Surprisingly, neither of these enzymes nor their combination was able to deglycosylate HrCHT1b, although ovalbumin was deglycosylated in same conditions (Figure S2). According to previous reports, some of the plant glycoproteins are resistant to enzymatic deglycosylation. It was shown that horseradish peroxidase isoenzyme-C, which contains eight N-linked glycans, was resistant to enzymatic deglycosylation by endoglycosidases [27]. PNGase F has broader specificity and is capable of hydrolysing amide of the asparagine (Asn) side chain only and any protein having N-glycans with fucose linked α-(1→3) to the Asn-bound N-acetylglucosamine, is resistant to PNGase F action. O-glycosidase, on the other hand, is specific for α-GalNAc linkages, however, glycoproteins with the absence of galactose or the acetamido group on the innermost N-acetyl-galactosamine residue prevents the action of O-glycosidase due to steric hindrance. In addition, any modification of the core structure can block the action of O-glycosidase. If the O-glycan structure is larger than the core structure, for example substituted with N-acetylglucosamine, N-acetylgalactosamine, sialic acid, or fucose, O-glycosidase will not cleave the GalNAc to Ser/Thr linkage. Therefore, resistance of HrCHT1b to enzymatic deglycosylation could be either due to presence of fucose linked α-(1→3) at the innermost GlcNAc, which is unique to plant glycans or the presence of O-glycan, larger than the core structure.

**Figure 5:**
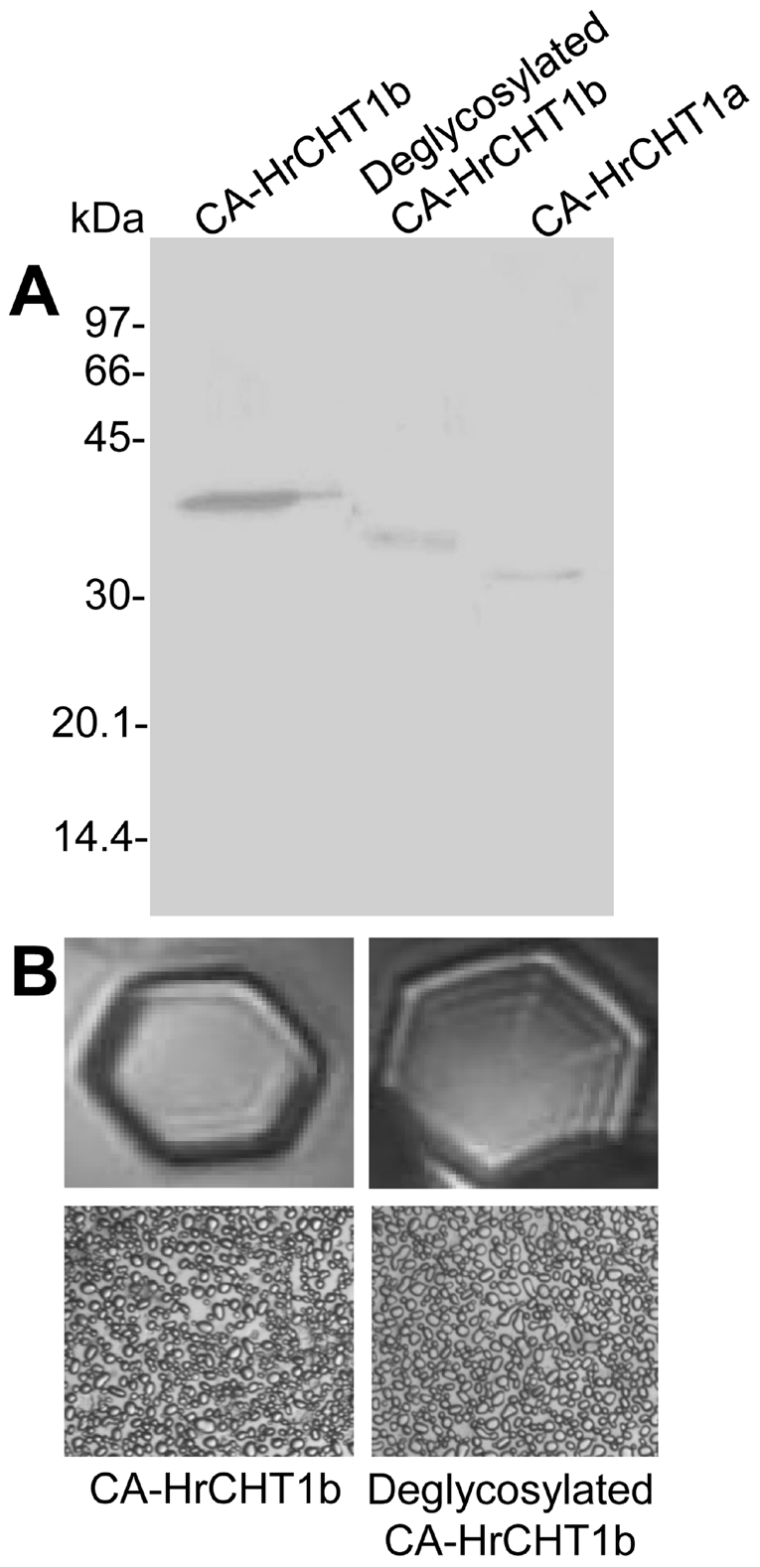
Deglycosylation of HrCHT1b. (A) SDS-PAGE profile showing deglycosylation of HrCHT1b using trifluromethanesulphonic acid. Antifreeze activity analysis of the glycosylated and deglycosylated HrCHT1b (B) by observing ice crystal morphology (upper panel) and ice recrystallization inhibition (lower panel).

As HrCHT1b was resistant to enzymatic deglycosylation, a chemical method using trifluromethanesulphonic acid was used to deglycosylate HrCHT1b. After chemical deglycosylation of HrCHT1b using TFMS, a gel shift from 34 to 32.5 kDa was observed on SDS-PAGE. As the size of deglycosylated HrCHT1b (32.5 kDa) was different than the size of HrCHT1a (31 kDa), it was concluded that the HrCHT1a was not the deglycosylated form of HrCHT1b (Figure 5).

To analyse the role of glycosylation in regulation of antifreeze activity, deglycosylated HrCHT1b was tested for its antifreeze activity which showed insignificant difference in the IRI (student’s t-test, *p*>0.05) between glycosylated and deglycosylated HrCHT1b. This suggests that glycosylation does not play any role in the regulation of antifreeze activity of HrCHT1b. Till date, antifreeze glycoproteins in plants are purified from *Solanum dulcamara*, carrot, *Lolium perenne* and seabuckthorn out of which, only *S. dulcamara* AFP showed glycosylation dependent antifreeze activity [11]. Glycosylation of HrCHT1b may have other roles, like in its targeting to apoplast or in protein stabilization.

### Calcium Differentially Regulates Hydrolytic and Antifreeze Activities of Chitinase

**Figure 6:**
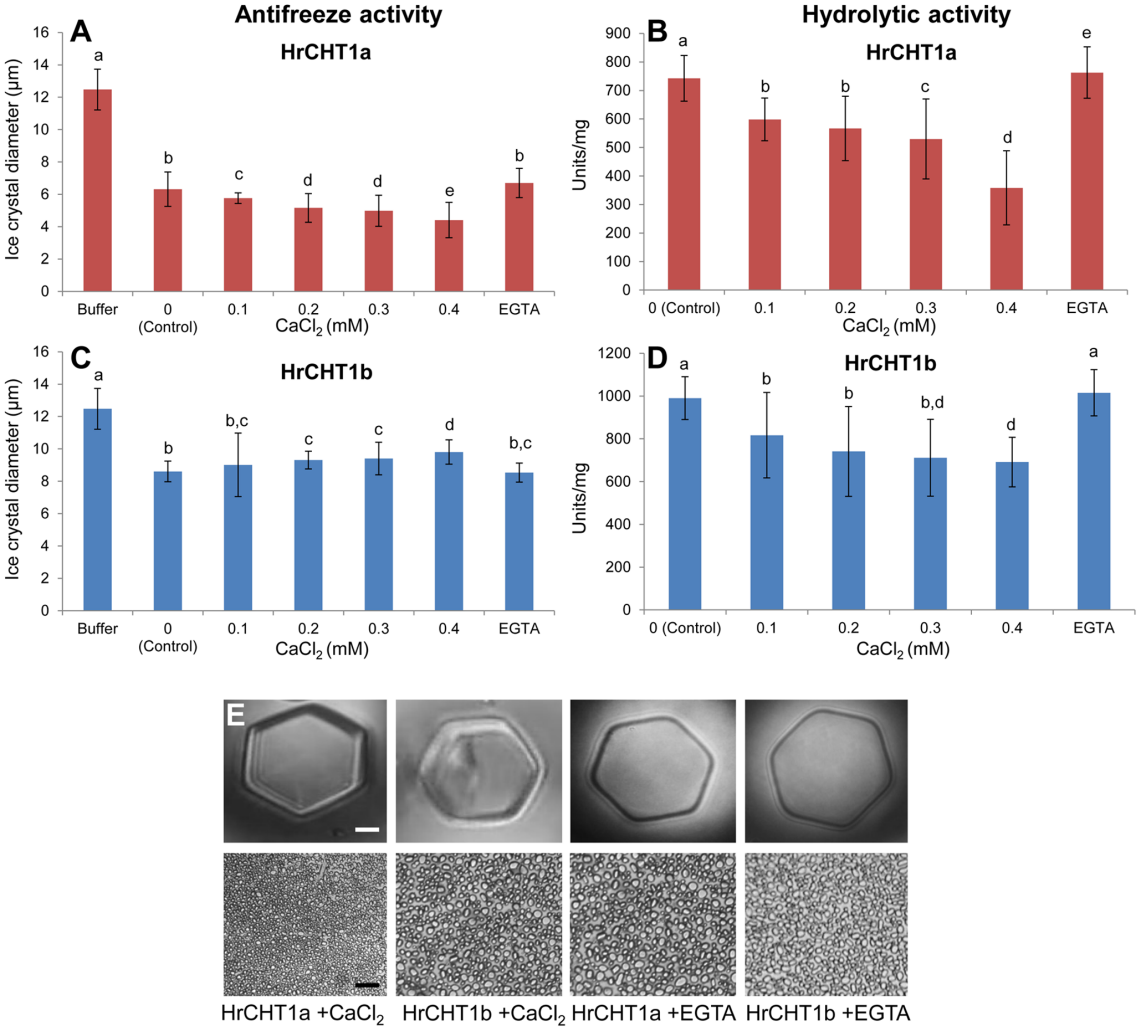
Analysis of effect of calcium on HrCHT1a and HrCHT1b. Effect of calcium on hydrolytic (A) and antifreeze activities (B, C) of HrCHT1a and HrCHT1b. Lower values of ice crystals diameter represent higher ice recrystallization inhibition and thus higher antifreeze activity. Error bars show standard deviation from three replicates. Statistical significance was determined by one-way ANOVA followed by Tukey−Kramer multiple comparisons test. Values with the same letters are not significantly different (*p*>0.05). Magnification bar in nanoliter osmometry shows 10 µm, while in splat assay it represents 50 µm.

Our results showed an enhancement in the antifreeze activity and reduction in the hydrolytic activity of HrCHT1a and HrCHT1b during cold acclimation. It is also well documented that calcium concentration increases during cold acclimation [28]. Therefore, to analyse any possible role of calcium in regulation of dual functionality of HrCHT1a and HrCHT1b, purified chitinases were incubated with CaCl_2_ (0–0.4 mM) and their associated activities were analysed. Interestingly, both the activities of HrCHT1a and HrCHT1b were affected by calcium. In case of HrCHT1a, an increase in the antifreeze (upto 14%) and decrease in its hydrolytic activity (upto 52%) was observed with calcium. However, in case of HrCHT1b, calcium decreased antifreeze (upto 30%) and hydrolytic (upto 33%) activity at 0.4 mM. The effect was partially reversed by the addition of 0.2 mM EGTA (Figure 6). These results showed that calcium also regulate the associated activities of HrCHT1a and HrCHT1b.

### Both HrCHT1b and HrCHT1a are Rich in β-stranded Conformation

**Figure 7:**
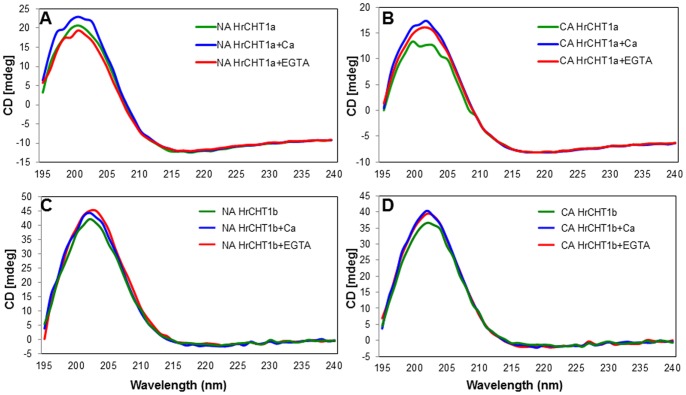
Secondary structure analysis of HrCHT1a and HrCHT1b. Far UV range CD spectra of HrCHT1a (A, B) and HrCHT1b (C, D) purified from non-acclimated (NA) and cold acclimated (CA) seedlings with 0.4 mM calcium and 0.2 mM EGTA. Each spectra presented in the figure are averaged from three spectra.

To analyse any change in the secondary structures of HrCHT1a and HrCHT1b during cold acclimation and in the presence of calcium, purified HrCHT1a and HrCHT1b were subjected to circular dichroism. CD results showed that HrCHT1a and HrCHT1b both had unusual type of highly *β*-stranded structure. Quantitative analysis of the CD spectra, using k2D3 server showed that HrCHT1a was composed of 36.47% *β*-strand and 4.51% *α*-helix while HrCHT1b was composed of 40.37% *β*-strand and 2.85% *α*-helix when purified from non-acclimated seedlings. When isolated from cold acclimated seedlings, HrCHT1a showed 39.39% *β*-strand and 2.37% *α*-helix while HrCHT1b showed 43.55% *β*-strand and 1.08% *α*-helix (Table 1). These results showed that chitinase isolated from cold acclimated seedlings showed higher percentage of *β*-strand and lower percentage of *α*-helix in comparison with those isolated from non-acclimated seedlings showing that these chitinases undergo refolding during cold acclimation. Interestingly, calcium also increases the percentage of *β*-strand both in HrCHT1a and HrCHT1b, suggesting that these conformational changes might be responsible for the calcium induced changes in the activities of these proteins (Figure 7). These results suggest that the dual functionality of the class I chitinases is regulated by changing their secondary structures and calcium seems to be an important factor in this regulation. From these results it can be concluded that the main function of HrCHT1a is antifreeze activity and that of HrCHT1b is hydrolytic activity. During cold acclimation, calcium is released from the calciosomes (most probably cell wall in this case) which enhances the antifreeze activity of HrCHT1a and inhibits its hydrolytic activity. At this point, HrCHT1a participates in cold stress tolerance by exhibiting antifreeze activity and HrCHT1b provide tolerance to psychrophilic pathogens by exhibiting hydrolytic activity. During non-acclimated conditions, calcium levels are low due to which HrCHT1a and HrCHT1b maintain their high hydrolytic activity to prevent the plant from fungal attack.

**Table 1:** Quantitative analysis of CD spectra of HrCHT1a and HrCHT1b.

S. No	Protein/treatment	Percentage of α-helix	Percentage of β-strand
**1**	NA-HrCHT1a	4.51	36.47
**2**	NA-HrCHT1a+Ca	3.84	37.9
**3**	NA-HrCHT1a+EGTA	3.86	37.85
**4**	CA-HrCHT1a	2.37	39.39
**5**	CA-HrCHT1a+Ca	0.92	41.9
**6**	CA-HrCHT1a+EGTA	1.30	41.35
**7**	NA-HrCHT1b	2.85	40.37
**8**	NA-HrCHT1b+Ca	1.37	41.73
**9**	NA-HrCHT1b+EGTA	1.59	42.06
**10**	CA-HrCHT1b	1.08	43.55
**11**	CA-HrCHT1b+Ca	1.01	43.39
**12**	CA-HrCHT1b+EGTA	1.01	43.29

The percentage of α-helix and β-strand in HrCHT1a and HrCHT1b isolated from non-acclimated (NA) and cold acclimated (CA) seedlings were calculated using K2D3 server, in presence or absence of calcium.

CD results showed that both HrCHT1a and HrCHT1b were rich in *β*-stranded conformation. To date, crystal structures of nine AFPs (one is for *L. perenne*, one for arctic yeast, four from insects and three from fishes) have been resolved. Out of these nine AFPs, seven including type III AFPs of fishes have *β*-sheet structure. Insect AFPs which are composed of *β*–sheets include *Cf*AFP from *Choristoneura fumiferana* (spruce budworm) and *Tm*AFP from *Tenebrio molitor* (yellow mealworm beetles). Crystal structure of *Ri*AFP (*Rhagium inquisitor* AFP), the most potent AFP known till date, is composed of a novel *β*-solenoid structure in which polypeptide chain forms a *β*-sandwich from parallel 6 and 7 stranded *β*-sheets [29]. Recently, structural analysis of glycosylated and non-glycosylated ice binding protein of Arctic yeast *Leucosporidium* sp. also showed that these are majorly composed of dimeric right-handed *β*-helix fold [30]. In case of plants, structure AFP from *L. perenne* was resolved recently using X-ray crystallography. *Lp*IBP (*L. perenne* ice binding protein) is composed of left handed *β*-roll and ice binding sites are formed of flat *β*-sheet present on one side of the *β*-roll [31]. In addition, high amount of *β*-sheeted structure has also been proposed for carrot AFP [32]. These results support existence of *β*-sheeted AFPs across the kingdoms. All these observations strengthen our finding that seabuckthorn chitinases are authentic AFPs but further structural details are required to analyse the ice binding sites of these chitinases.

CD results showed that both the chitinases undergo conformational changes during cold acclimation and in the presence of calcium. There are previous studies which showed refolding of AFPs during low temperature or in the presence of ice. AFP from *Dendroides canadensis* is composed of ∼46% *β*-sheet, 39% turn, 2% helix and 13% random structure at 25°C. Binding of AFP to the ice crystal led to an increase in *β*-sheet and helix content at the expense of turn and random structure [33]. Moreover, type I AFP has been shown to undergo helix-coil transition. At 0°C, type I AFP is 50% *α*-helical while this helicity is lost at 22°C [34]. Spruce budworm AFP increases in *β*-strand conformation during lowering of temperature from 30°C to –5°C [35]. In addition, AFGP, in which *β*-conformation seems to be dominating, refolds during low temperature which may expose ice binding residues [36]. CD results also showed that both HrCHT1a and HrCHT1b refold in the presence of calcium. Structural changes in class I chitinase with calcium was also observed in winter rye [15] where freezing and thawing of chitinase in the presence of calcium results in formation of new calcium binding sites. Calcium binds with these sites and increases the hydrolytic activity. Effect of calcium on antifreeze activity of class I chitinase was not analysed previously.

During cold acclimation or in the presence of calcium, the *α*-helical content of chitinase decreases and its *β*-stranded conformation increases which is responsible for changes in the activities of these two proteins. Similar kind of *α*-helix to *β*-strand conversion has been shown during or after formation of amyloid fibrils in animals. Amyloid fibrils are insoluble fibrous proteins which are associated with many diseases including Alzheimer’s disease. Amyloid forming proteins are made up of *α*-helix while amyloid fibrils are made up of *β*-strands perpendicular and *β*-sheets parallel to the fiber axis [37]. Therefore these undergo α→β conversion at the time of fibrils formation. This α→β conversion of proteins is a spontaneous and non-enzymatic reaction which occurs mainly due to rearrangement of aspartic acid or asparagine residue particularly in the tertiary regions in Asp-Gly, Asn-Ser, Asn-Gly and Asn-His [38]. However, in animals, this α→β conversion of proteins and formation of amyloid fibrils results in protein aggregation which results in loss of activity and function of these proteins. Therefore, AFPs must possess some mechanism to prevent this aggregation and to prevent its activities. In *Ri*AFP, it has been shown that at the C-terminal, *β*-strands form a cap which prevents end to end interaction and thus aggregation of *Ri*AFP. In addition, presence of three bulky groups (two glutamine and one isoleucine) at the C-terminal also participates in capping and thus prevents amyloid-like polymerization. AFPs have been shown to bind with the ice crystals by a flat ice binding surface which could be the reason of absence of twist in the AFPs structure.

### Unfolding Kinetics of HrCHT1b and HrCHT1a

**Figure 8:**
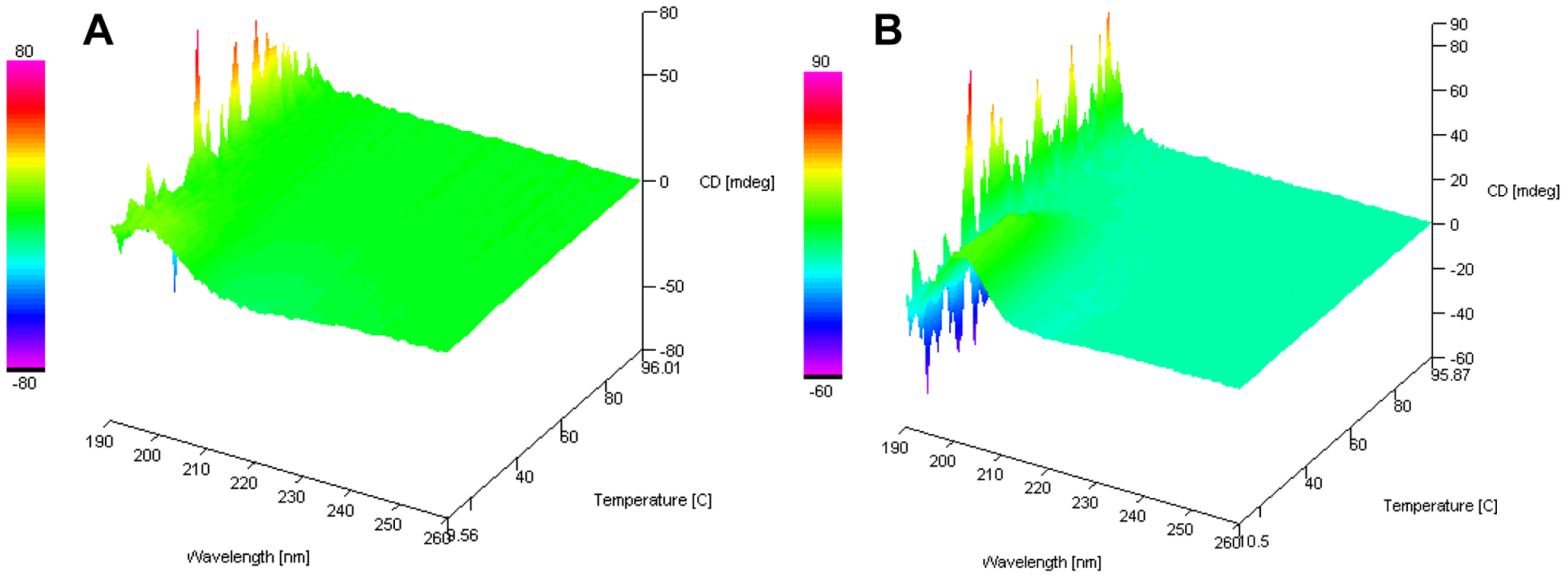
Analysis of unfolding kinetics of HrCHT1a and HrCHT1b. Far UV range CD spectra of HrCHT1a (A) and HrCHT1b (B) from temperature 10°C to 94°C at an interval of every 2°C.

CD results showed that chitinase undergo α→β conversion during cold acclimation. To predict whether this change is reversible and only low temperature is responsible for this change, HrCHT1a and HrCHT1b, purified from cold acclimated seedlings were heated upto 94°C and the kinetics of chitinase folding was observed using circular dichroism. The spectra for each chitinase were acquired after every 2°C. If the α→β conversion of HrCHT1b and HrCHT1a was due to lower temperature only, these must undergo β→α conversion at higher temperatures. Analysis of the CD spectra showed that neither HrCHT1a nor HrCHT1b undergoes β→α conversion at elevated temperatures showing that this α→β conversion of HrCHT1b and HrCHT1a was irreversible. However this seems to be highly unlikely in terms of energy expenditure that this α→β conversion of chitinases is irreversible as this would lead to formation of new chitinase proteins after the cold stress is over. At this point of time it is difficult to explain why this is irreversible. However, it is quite possible that in *in vivo* conditions, protein chaperonins help in proper refolding of these chitinases after stress. In addition CD spectra also showed that HrCHT1a started degrading at 30°C and completely degraded at 40°C while HrCHT1b started unfolding at 40°C and completely degraded at 60°C, unlike other chitinases and AFPs some of which are boiling stable (Figure 8).

## Conclusion

Not much is known about the regulation of class I chitinase with respect to its dual functionality. Previous reports suggested that chitinase acquire antifreeze activity during cold acclimation. Notwithstanding, we have shown that antifreeze and hydrolytic activities are the constitutive properties of class I chitinase which alter during cold acclimation. During cold acclimation, hydrolytic activity of the HrCHT1a and HrCHT1 decreases while their antifreeze activity increases. Like insect and *L. perenne* AFPs, both HrCHT1a and HrCHT1b are *β*-stranded. Besides, we have shown that HrCHT1a and HrCHT1b undergoes α→β conversion during cold acclimation which might be responsible for the reduction of hydrolytic and enhancement of antifreeze activity. These results suggest that besides promoting protein accumulation, cold also causes structural changes, both of which contributes to cold stress tolerance. Moreover, calcium regulates both the activities of the two chitinases by changing the secondary structure. These results shed a light on the regulation of class I chitinase and showed that antifreeze and hydrolytic activities are the constitutive properties of class I chitinase and calcium and cold fine tunes these activities by changing the secondary structure.

## Supporting Information

Figure S1 **SDS-PAGE gel showing elution of HrCHT1a and HrHT1b using different concentrations of acetic acid (15–250 mM).** In lane 1, chitin beads after washing with 20 mM ammonium bicarbonate were dissolved in sample buffer and directly loaded on the gel.
(TIF)

Figure S2 **SDS-PAGE showing deglycosylation of ovalbumin and HrCHT1b attempted using PNGase F, O-glycosidase and their combination.**
(TIF)
