# Supplementary figures and images for: Refolding of β-Stranded Class I Chitinases of Hippophae rhamnoides Enhances the Antifreeze Activity during Cold Acclimation
Source: PLoS One. 2014 Mar 13;9(3):e91723. doi: 10.1371/journal.pone.0091723 (PMC3953593; doi:10.1371/journal.pone.0091723)

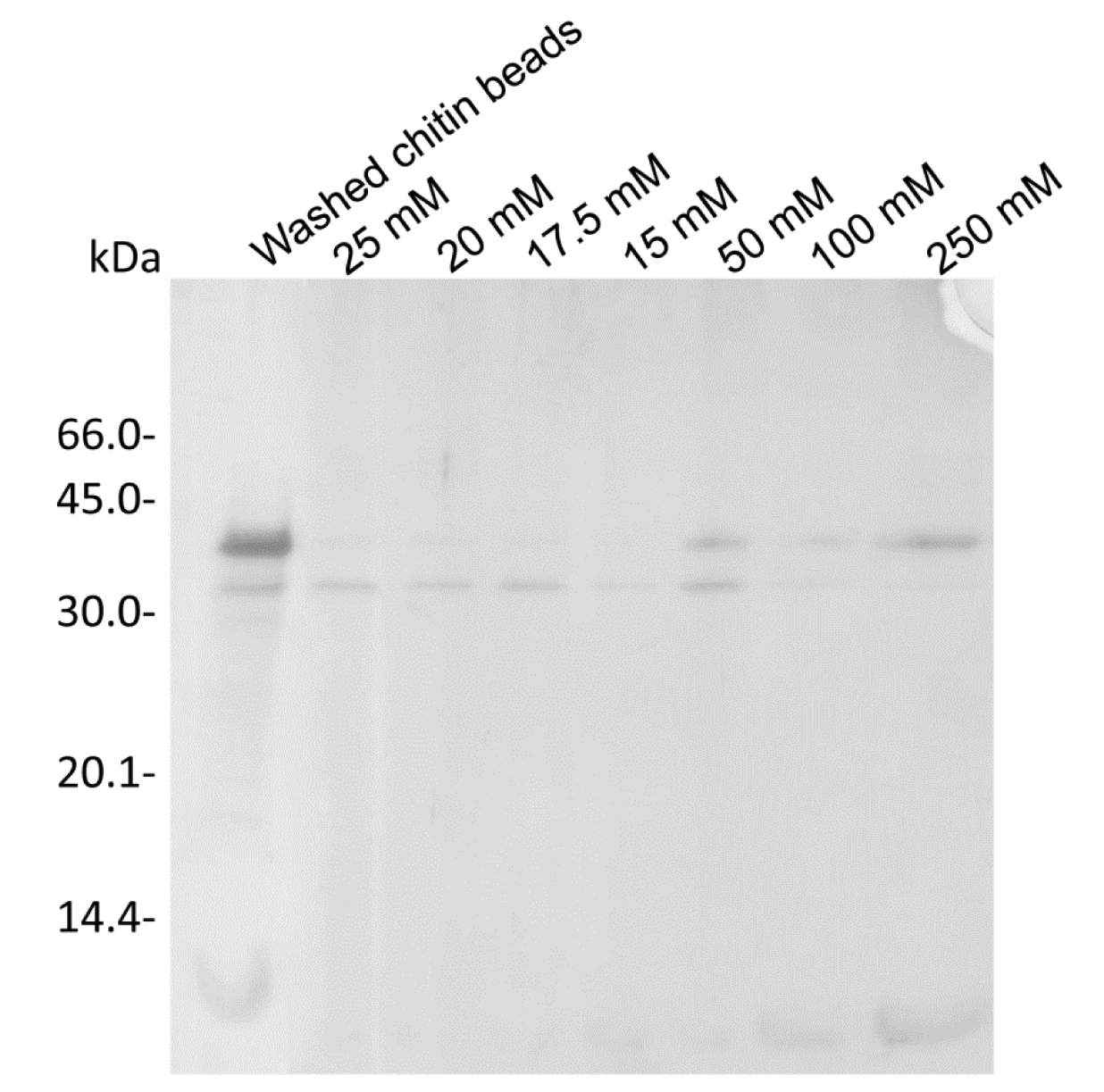

Supplement: Figure S1 — SDS-PAGE gel showing elution of HrCHT1a and HrHT1b using different concentrations of acetic acid (15–250 mM). In lane 1, chitin beads after washing with 20 mM ammonium bicarbonate were dissolved in sample buffer and directly loaded on the gel. (TIF) [file pone.0091723.s001.tif]

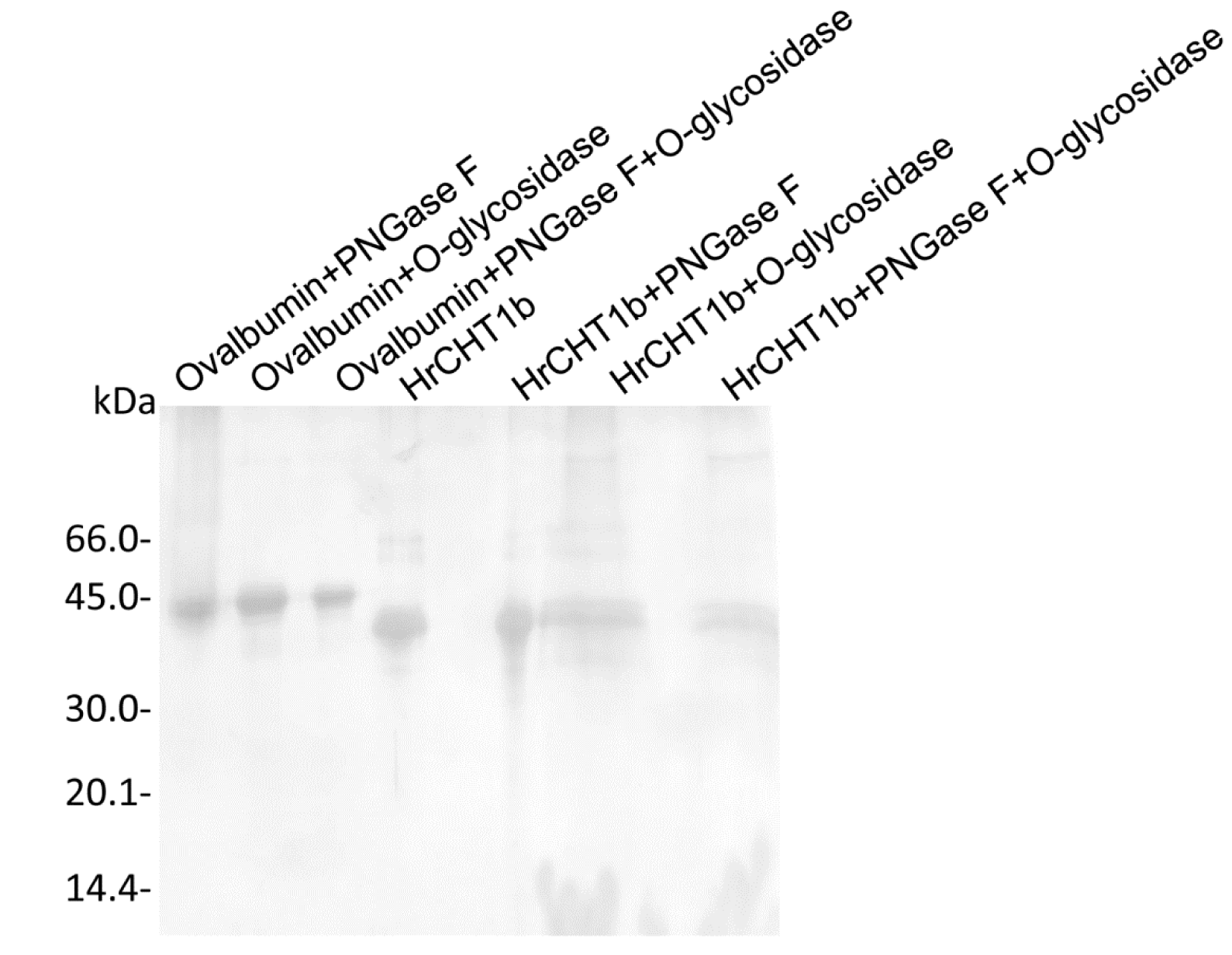

Supplement: Figure S2 — SDS-PAGE showing deglycosylation of ovalbumin and HrCHT1b attempted using PNGase F, O-glycosidase and their combination. (TIF) [file pone.0091723.s002.tif]
